# Unsupervised anomaly detection with generative adversarial networks in mammography

**DOI:** 10.1038/s41598-023-29521-z

**Published:** 2023-02-20

**Authors:** Seungju Park, Kyung Hwa Lee, Beomseok Ko, Namkug Kim

**Affiliations:** 1 Department of Biomedical Engineering, College of Health Sciences, Korea University, Seoul, Republic of Korea; 2 Department of Radiation Oncology, Korea University Guro Hospital, Korea University College of Medicine, Seoul, Republic of Korea; 3 Department of Breast Surgery, University of Ulsan College of Medicine and Asan Medical Center, 88 Olympic-ro 43-gil Songpa-gu, Seoul, 05505 Republic of Korea; 4 Department of Radiology, University of Ulsan College of Medicine and Asan Medical Center, Seoul, Republic of Korea; 5 Department of Convergence Medicine, Research Institute of Radiology and Institute of Biomedical Engineering, University of Ulsan College of Medicine and Asan Medical Center, 88 Olympic-ro 43-gil Songpa-gu, Seoul, 05505 Republic of Korea

**Keywords:** Cancer, Computational biology and bioinformatics, Engineering

## Abstract

Breast cancer is a common cancer among women, and screening mammography is the primary tool for diagnosing this condition. Recent advancements in deep-learning technologies have triggered the implementation of research studies via mammography. Semi-supervised or unsupervised methods are often used to overcome the limitations of supervised learning, such as manpower and time, for labeling in clinical situations where abnormal data are significantly lacking. Accordingly, we proposed a generative model that uses a state-of-the-art generative network (StyleGAN2) to create high-quality synthetic mammographic images and an anomaly detection method to detect breast cancer on mammograms in unsupervised methods. The generation model was trained via only normal mammograms and breast cancer classification was performed via anomaly detection using 50 breast cancer and 50 normal mammograms that did not overlap with the dataset for generative model learning. Our generative model has shown comparable fidelity to real images, and the anomaly detection method via this generative model showed high sensitivity, demonstrating its potential for breast cancer screening. This method could differentiate between normal and cancer-positive mammogram and help overcome the weakness of current supervised methods.

## Introduction

Breast cancer is the most commonly diagnosed cancer and the leading cause of cancer death among women^1,2^. Mammography is regarded as the most effective screening tool for breast cancer detection and diagnosis. Screening mammography has been shown to reduce the rate of death from breast cancer by 25% in women between the ages of 50 and 69 years based on the results of several randomized clinical trials^3–6^. Recent studies have observed reductions in breast cancer mortality in service screening programs consistent with those observed in the randomized trials, although the use of screening mammography remains controversial due to concerns regarding methodological limitations in some of the randomized trials^7,8^. Moreover, Kalager et al. investigated the availability of screening mammography via valid comparison groups instead of historical control participants to consider chronologic trends associated with advances in breast cancer awareness and treatment^9^. The authors concluded that screening mammography was still associated with a reduction in the rate of death from breast cancer, but screening itself accounted for only about a third of the total reduction.

Low contrast between cancerous lesion and normal breast tissues is one of the most significant challenges of mammography, which makes it difficult for radiologists to interpret the results. Computer-aided diagnosis and detection of abnormalities in mammography have been introduced and play an important role in breast cancer screening^10,11^. Furthermore, recent advances in machine learning and deep-learning (DL) networks have become powerful techniques by enabling automatic feature extraction and detection in various fields as well as in medical images^12–16^. Recent studies using DL methods, specifically convolutional neural networks with supervised learning, improved the ability of radiologists to detect even the smallest breast cancers at their earliest stages, thus alerting radiologists when further analysis is needed^17–22^. Despite the superior performance, supervised methods are vulnerable in evaluating data that are completely different from the data the model encounters during training. In addition, labeling large amounts of training data for supervised learning requires enormous manpower and time resources. Several studies with unsupervised training have been introduced to alleviate the burden of manual annotation^23,24^.

Recently, various DL-based generation models for high-resolution images have been introduced^25–31^. Especially, generative adversarial networks (GANs) have been developed and improved the synthetic performance for high-resolution images^27^. Several studies have applied GAN to generate realistic medical images from various imaging modalities, such as X-ray, computed tomography (CT), and magnetic resonance imaging^32–35^. Furthermore, studies have been published that not only generate medical images using GAN but also use these synthesized images for data augmentation or apply them to anomaly detection^36–40^. In the current study, we generated realistic normal mammographic images using the state-of-the-art generation network StyleGAN2^31^ and developed an unsupervised anomaly detection method to detect breast cancer without the need to collect or annotate cancer datasets.

## Results

**Figure 1:**
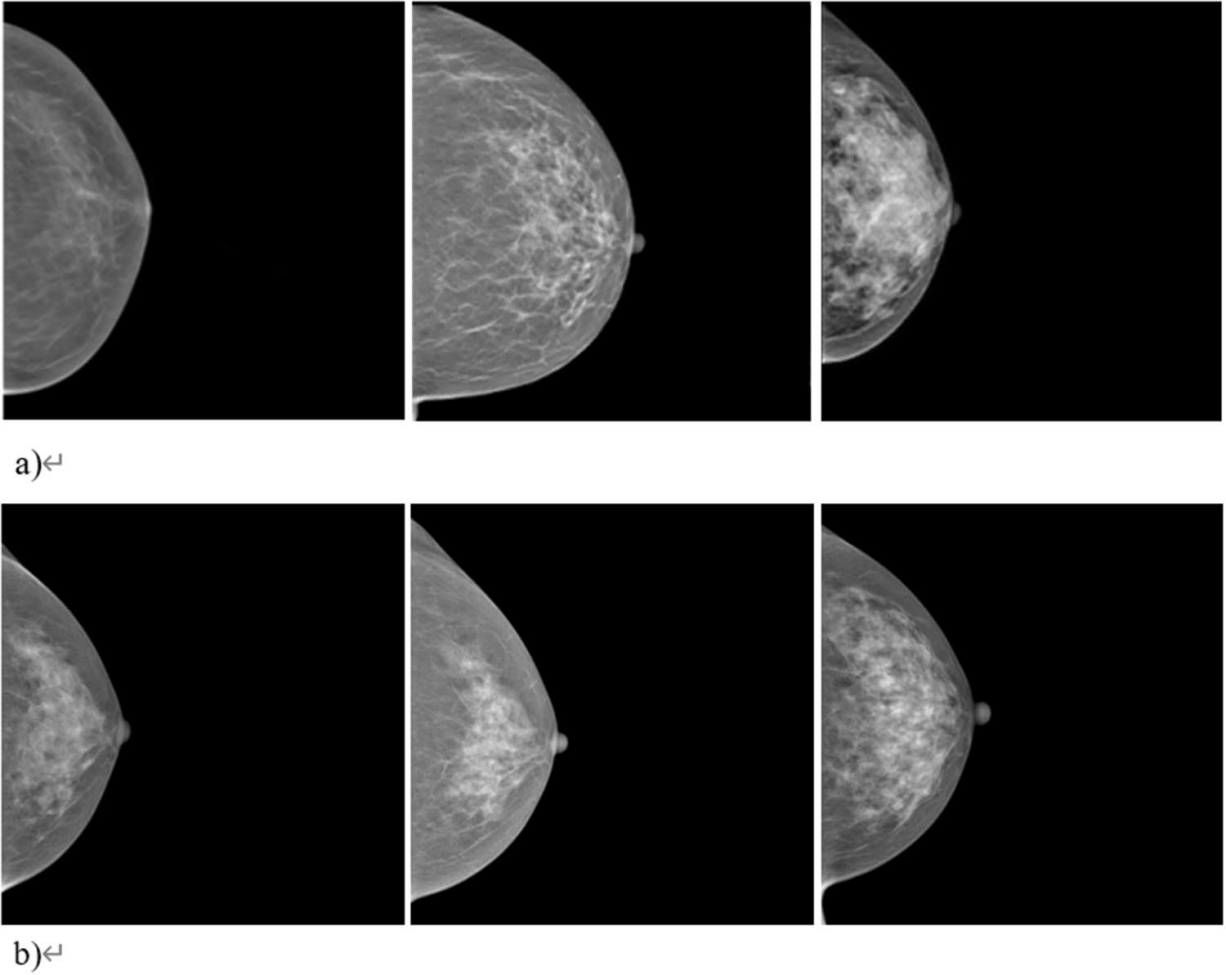
Examples of generated images from the StyleGAN2 model. Each Frechet inception distance score is 10.425, and 4.383 for (**a**) and (**b**), respectively.

**Figure 2:**
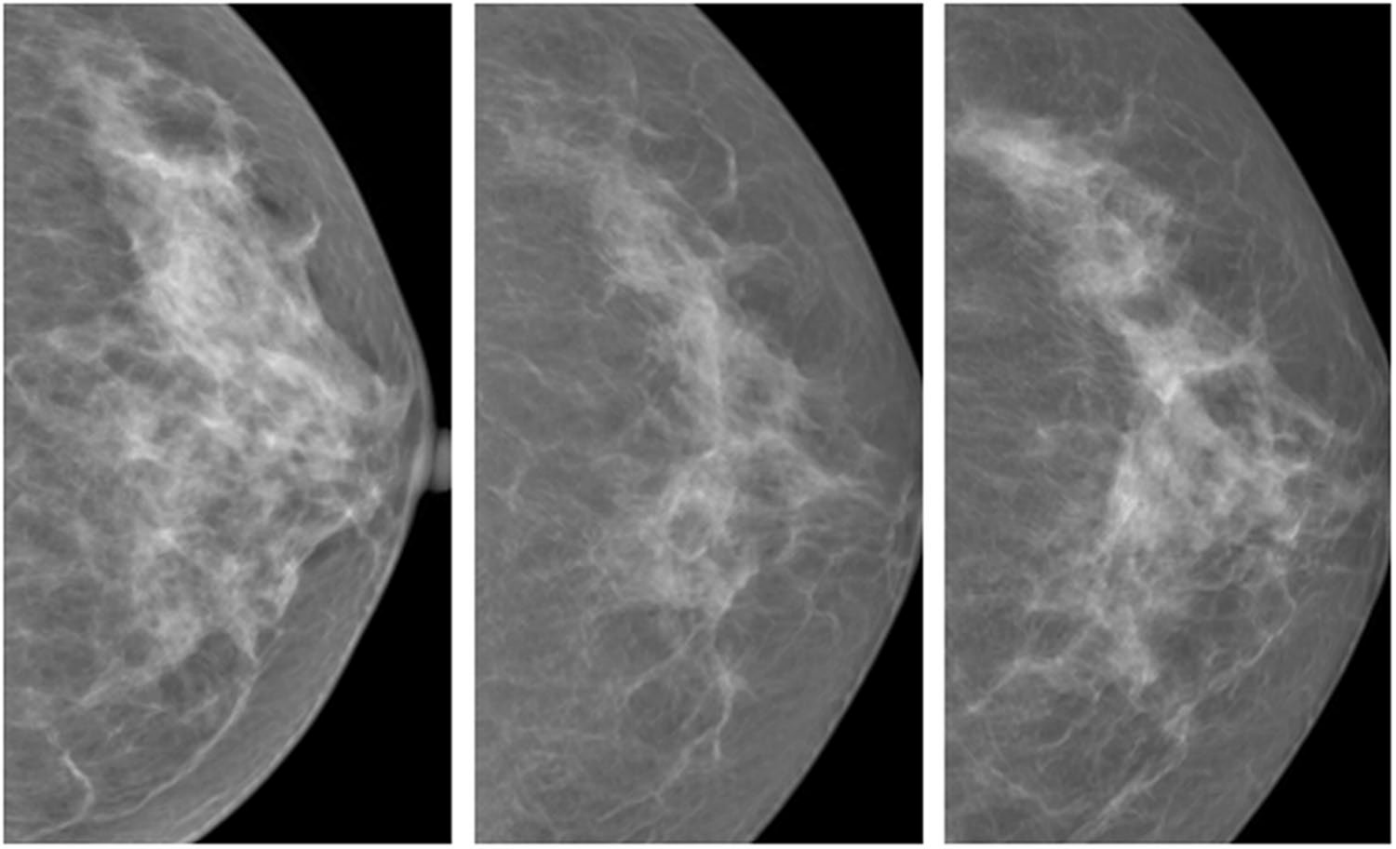
Unusual noise-like patterns in the synthesized mammographic images.

Figure 1 shows the examples of generated normal mammographic images from the styleGAN2 model. The best Frechet inception distance (FID)^41^ and inception score^42^ were 4.383 and 16.67, respectively. The multiscale structural similarity for image quality assessment (MS-SSIM)^43^ and average value of peak signal-to-noise ratio (PSNR)^44^ of the synthesized images were 0.39 and 31.35, respectively. The overall breast morphologies and internal parenchymal structures of synthetic images were highly realistic. However, unusual noise-like patterns were noticed inside the parenchymal structure of the breast in the magnified view, which were not identified in real mammographic images (Fig. 2).

**Figure 3:**
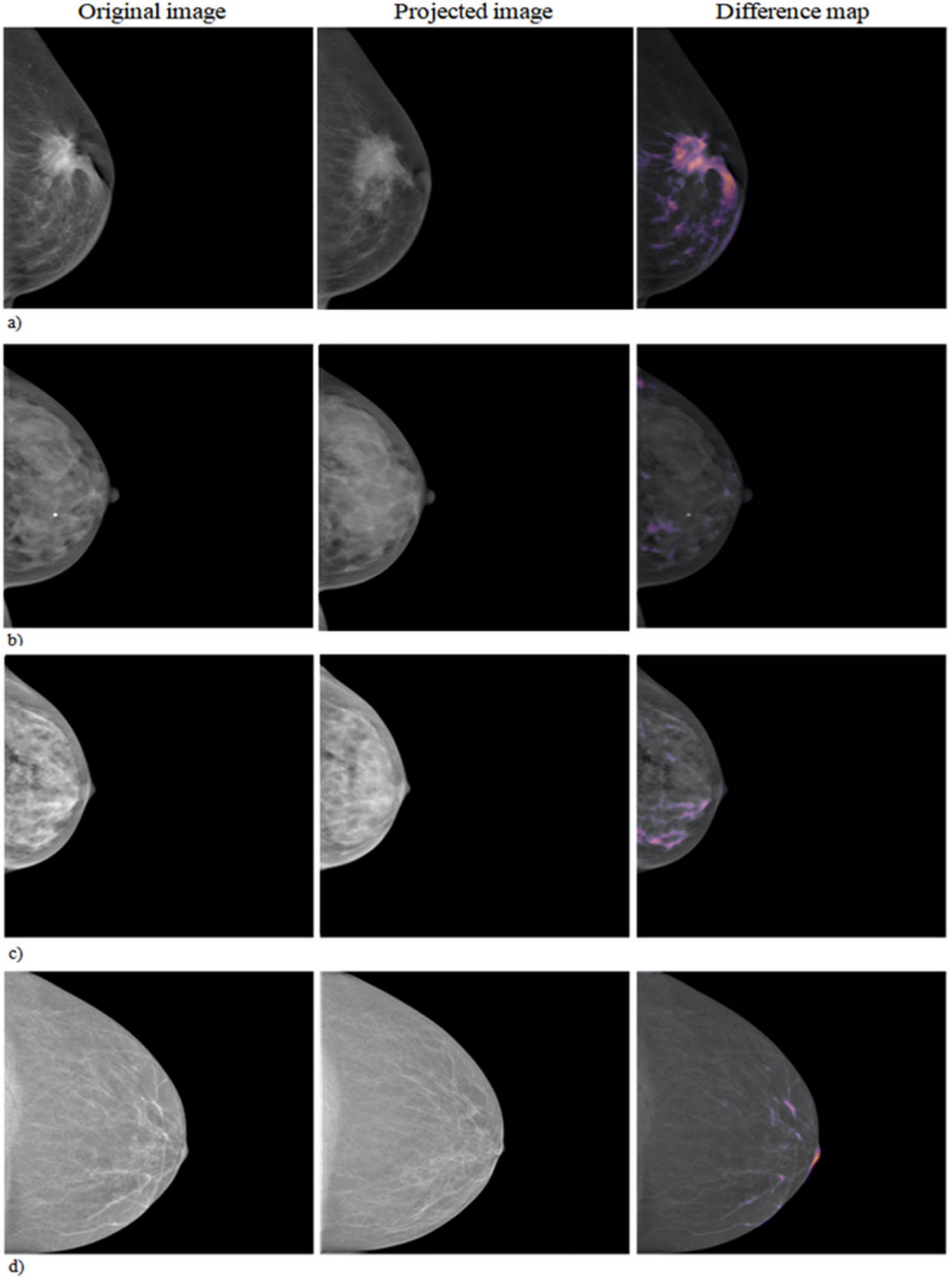
Examples of anomaly detection with real mammographic image as an input (first image), one of the most similar nine normal synthetic mammographic images as an output (second image), and difference map between the real image and the synthetic image (last image). (**a**–**d**) Show true-positive, false-negative, false-positive, and true-negative case in order.

Figure 3 shows cases of true-positive (TP), false-negative (FN), false-positive (FP), and true-negative (TN) of breast cancer detection using anomaly detection method. For each of the four cases depicted, the images in each row represent the real image, one of the nine synthetic images that was most similar to the real image (projected image), and the difference map between the real image and the average of nine synthetic images.

**Figure 4:**
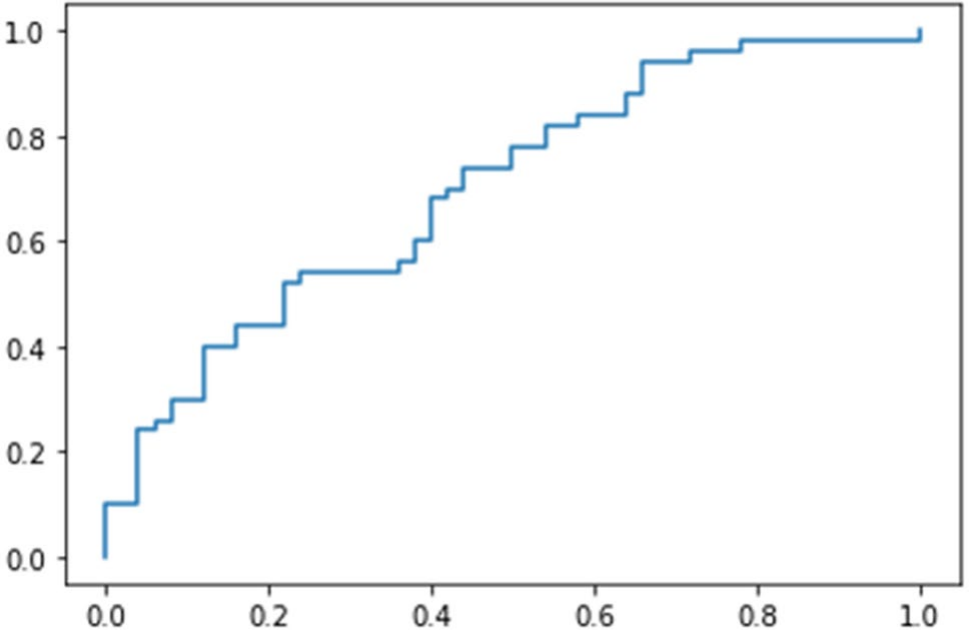
The receiver operating characteristic curve of classification for breast cancer.

**Figure 5:**
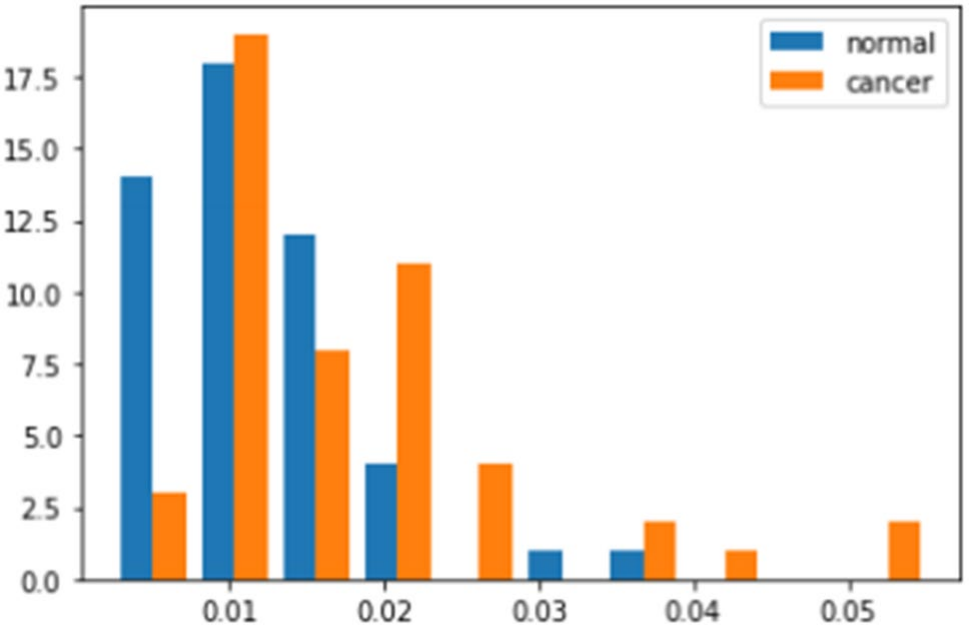
Histogram for anomaly scores.

Table 1 shows the classification results for breast cancer using anomaly detection method in according to the number of synthetic image seeds created per image. The use of nine different seeds provided the highest performance with accuracy, sensitivity, specificity, positive predictive value (PPV), and negative predictive value (NPV), and the areas under the receiver operating characteristic (ROC) curve (AUC) were 64.0%, 78.0%, 52.0%, 61.4%, 70.2%, and 70.0%, respectively. Figure 4 demonstrates a ROC curve of classification performance for breast cancer. A histogram of anomaly scores in breast cancer and normal patients is depicted in Fig. 5.

**Table 1:** Comparisons of performance on classification for breast cancer with the number of seeds per image.

No.^1^	Accuracy	Sensitivity	Specificity	PPV	NPV	AUC
1	61.0	74.0	48.0	58.7	64.9	66.6
9	64.0	78.0	52.0	61.4	70.2	70.0
16	63.0	78.0	48.0	60.0	68.6	67.8

^1^Number of seeds of synthetic images created per image.

Data are presented as n (%). *PPV* positive predictive value, *NPV* negative predictive value, *AUC* area under the receiver operating characteristic curve.

## Discussion

In this study, we generated highly realistic mammographic images with a state-of-the-art DL-based generative network using normal mammograms and developed an unsupervised anomaly detection method for breast cancer detection. The generation performance was measured as FID score of 4.383 and inception score of 16.67, respectively. The AUC, sensitivity, and specificity of the classification performance for breast cancer detection were 70.0%, 78.0%, and 52.0%, respectively.

Recently, DL-based various generation models were introduced. In particular, as GANs were published in 2014^45^, they have been a hot research topic, and many strategies and GAN variants have been proposed^28,30,31,46^. GANs consist of two neural networks, i.e., generator and discriminator networks. The generator produces synthetic images from random noise vectors and tries to fool the discriminator, whereas the discriminator tries to distinguish the fake samples from the real samples. Kerras et al. demonstrated that the style-based generator architecture for GANs (StyleGAN) was very effective in generating high-resolution images by learning both global attributes and stochastic details^30^. The StyleGAN model mapped the latent space Z into the W space via a nonlinear mapping network and then merged into the synthesis network via adaptive instance normalization at each convolutional layer. In the StyleGAN2 model, the authors redesigned the generator normalization, revisited progressive growing, and regularized the generator to encourage good conditioning in the mapping from latent codes to images to remove characteristic blob-shaped artefacts and improve image quality^31^. In the current study, the StyleGAN2 model was used to generate synthetic mammographic images. Optimal training was given through visual observation of synthesized mammographic images and monitoring of FID and inception scores while adjusting the learning rate. The model initially generated a coarse shape of the breast, and as training progressed, it generated the complex parenchymal tissues inside the breast.

GANs have been observed to suffer from mode collapse, in which the generator learns to generate examples from only a few modes of the data distribution and misses many other modes, even if examples of the missing modes exist throughout the training data^47,48^. In this study, we observed that the model falls into a mode collapse in which nothing is generated when training continues after the model shows optimal generation performance. The optimal training point was established through visual observation and monitoring the lowest FID score to use the unsupervised anomaly detection method using the generated normal mammographic images. Some synthesized images showed unusual noise-like patterns in parenchymal structure within the breast that were not identified in real mammographic images, although most of the generated images showed similar fidelity to real mammographic images. In the StyleGAN2 architecture, geological features are learned from coarse-to-fine scale through a progressive training process^31^ in which per-pixel noise was injected after each convolution to compensate for the loss of information compression, thereby capturing high-variance details. The unusual noise-like patterns might have been caused by the network structure. This suggests that the highly complex and variable parenchymal structures within the breast are too fine to be trained as a style and thus, may not be trained efficiently.

Anomaly refers to an observation that is significantly out of the concept of normality, and anomaly detection is a technique for detecting a state that is not normal^49^. For anomaly detection, a one-class classification method that learns a hypersphere so that general normal features gather at one point, or a feature matching method that determines an anomaly based on the distance or probability distribution of features can be used. Schlegl et al. first developed a method using GANs for anomaly detection (AnoGAN)^38^. The authors computed anomaly scores based on latent space mapping using a deep convolutional GAN trained only with normal data. This method has recently been applied as a method for finding disease-related anomalies in various medical images^39,40,50,51^. Lee et al. developed an anomaly detection algorithm with a deep generative model trained on brain CT images of healthy individuals for detecting emergency cases^50^. They demonstrated that the median wait time was significantly shorter, and the median radiology report turn-around time was significantly faster with their anomaly detection algorithm in a clinical simulation test of an emergency cohort. In this study, we developed an unsupervised anomaly detection method for detecting breast cancer using synthetic normal mammographic images with a deep generative model. To the best of our knowledge, this paper was the first study to classify breast cancer on mammographic images using unsupervised anomaly detection algorithm, demonstrating its preliminary results. Although the classification performance was not yet high enough and the validation dataset was small, our results showed the potential of the method to classify breast cancer using the unsupervised method.

Recently, several supervised DL-based studies for breast cancer detection in mammographic images have been introduced^10,19,21,22,52–55^. In their study, artificial intelligence (AI) algorithm showed better diagnostic performance in breast cancer detection compared with radiologists, and radiologists performed significantly better when assisted by AI^19,21,22,53–55^. Despite these superior performances, the supervised method has some limitations. First, it can be difficult for the network to classify new unseen data that has not been learned during training, even if it contains some artefacts that physicians can easily identify, resulting in the network inferring inaccurate results. Second, a large amount of annotated data is inevitably required. Particularly, abnormal medical data are usually scarce compared with normal data, and only trained medical experts can annotate data in most cases. Kim et al. collected annotated data involving over 150,000 mammograms, including breast cancers, benign masses, and normal breasts, in training the classification network in a supervised manner^21^. Semi-supervised or unsupervised methods were studied as useful alternatives to supervised methods to overcome drawbacks in a supervised learning^23,38–40^. In this study, we were able to detect breast cancer using an unsupervised method without having to collect and annotate large amounts of cancer datasets. This method could be used as an additional screening tool or alarm system to compensate for the deficiencies in supervised methods.

Results were compared according to the number of seeds used to generate the images to reduce the proportion of false-positive cases that are detected as cancer in areas with high parenchymal density in the breast, although no cancer is actually present. The best performance was achieved when nine different seeds were used, with AUC, sensitivity, and specificity of 70%, 78%, and 52%, respectively. This result indicates that using 1 seed may be relatively insufficient to remove false-positive regions, whereas using 16 seeds may result in poor performance when averaging difference maps due to the large number of different images. In addition, benign diseases were not excluded when mammographic images were collected in training the generation model, which may have led to an increase in false-negative cases as some benign masses look similar to cancer. To overcome these weaknesses, future studies could consider methods to improve the performance of projection on test images and stepwise models that can reduce the number of false-negative cases by first excluding benign masses. The staged model can be implemented by filtering abnormal cases using a model trained with only normal mammograms without benign masses followed by cancer cases using a model trained with only mammograms with benign masses.

This study had several limitations. First, only craniocaudal views of mammograms with limited resolution were used for the generation of images and detection of anomalies. Therefore, generation with mediolateral-oblique views of mammograms and full high-resolution images (i.e., 2294 × 1914 pixels), which are commonly used in the real clinical field, would allow for more accurate anomaly detection. Second, we could not conduct the image Turing test by radiologists to evaluate the qualitative performance of the generated images. Particularly in medical images, evaluation for qualitative performance might be more crucial than quantitative evaluation, which only measures differences in the density between two distributions from real image and fake image in the high-dimensional feature space. Finally, our preliminary results for breast cancer detection showed insufficient performance for clinical application. Therefore, improvements through more similar projections for cancer images and a staged generation model to distinguish benign cases should be considered to investigate its potential as an additional screening tool.

In conclusion, this study proposes a generative model that uses StyleGAN2 model for the generation of high-quality synthetic mammographic images and the anomaly detection method for the detection of breast cancer on mammograms in an unsupervised manner. Our generative model has shown comparable fidelity to real images, and the anomaly detection method via this generative model trained with only normal mammograms could differentiate between normal and cancer-positive mammograms. This method could provide additional information and help overcome the weakness of current supervised methods for breast cancer detection.

## Methods

### Data collection

We retrospectively reviewed electronic medical records of patients with breast cancer who underwent mammography in Asan Medical Center between January, 2008 and December, 2017. Normal mammograms were collected from mammograms of normal breast contralateral to cancer and their follow-up mammograms. Mammograms containing surgical clips were excluded from this study because the clip’s sudden high intensity could significantly adversely affect the generative performance of the GAN. Finally, this study included 105,948 normal mammograms from 22,848 patients for training the generation model. Only craniocaudal views of the mammographic images were used for model training. Additionally, we collected datasets to evaluate the anomaly detection method for breast cancer detection. Fifty mammograms of breast cancer, which were pathologically staged to T stages 1 to 4, according to the 8th edition of the American Joint Commission on Cancer Staging^56^, and 50 normal mammograms that did not overlap with those used to train the generative model were obtained. This retrospective study was conducted according to the principles of the Declaration of Helsinki and was performed in accordance with current scientific guidelines. The protocols of this study were approved by the Institutional Review Board of Asan Medical Center (IRB number: 2017-1341), and the requirement for informed consent from patients was waived due to the retrospective nature of the study.

### Generation of mammographic images

GANs consists of two neural networks, generator and discriminator networks, where the generator’s cost encourages it to generate samples that the discriminator incorrectly classifies as real, while the discriminator’s cost encourages it to correctly classify data as real or fake^27^. The process can be described as a minimum–maximum game shown in the following function (1):

1 $$ {\text{min}}_{{\text{G}}} {{\text{max}}}_{{{\text{D}}}} {{\text{V}}}({{\text{D,}}}\;{{\text{G}}}) = {{\text{E}}}_{{{{\text{x}}}\sim {{\text{P}}}_{{{{\text{data}}}({{\text{x}}})}} }} [\log {{\text{D}}}({{\text{x}}})] + {{\text{E}}}_{{{{\text{Z}}}\sim {{\text{P}}}_{{{{\text{z}}}({{\text{Z}}})}} }} [\log (1 - {{\text{D}}}({{\text{G}}}({{\text{z}}})))], $$

where x is a “real” sample from the actual dataset, represented by distribution P_data(x)_, and z is a “latent vector” sampled from the distribution P_z(Z)_, which is typically noise. The StyleGAN model mapped the latent space Z into the W space via a nonlinear mapping network (an eight-layer MLP) and then merged into the synthesis network via adaptive instance normalization (AdaIN) at each convolutional layer^57^. A gaussian noise is added after each convolutional layer before the AdaIN layer. AdaIN was restructured for weight demodulation in the StyleGAN2 model, and progressive growth was removed because it introduced small artefacts during image generation.

All mammograms of right-sided breasts were aligned to the left by flipping along their vertical axis to use only left-sided mammograms for training efficiency to train the StyleGAN2 model with mammographic images. The windowing level of each image was adjusted using a center and a width value of each image from digital imaging and communications in medicine (DICOM) header information to most closely match the image that doctors see in the image viewer. The original 12-bit grayscale DICOM images were converted into 8-bit grayscale. The size of the original image (2294 × 1914 pixels) was then changed to a modified size (2294 × 2294 pixels) by padding zeros on the right edge and downscaled to 512 × 512 pixels for training efficiency. A publicly available official implementation of StyleGAN2 via Tensorflow in Python was used. The model was trained using the original NVIDIA implementation on a computer with a Linux operating system. The learning rate and batch size were set at 0.001 and 8, respectively, and other parameters were fixed as default values while training. A quantitative analysis was conducted to evaluate the qualities of generated images. We used the FID that measures differences in density of between two distributions in the high-dimensional feature space of an InceptionV3^58^ classifier, which compares the activation of a pretrained classification network on real and generated images. In addition, the inception score, MS-SSIM, and PSNR were measured. MS-SSIM is used to measure the diversity of generated image, and the similarity between two images is computed based on image pixels and structures. The mean MS-SSIM score was measured between randomly selected pairs of synthetic-to-synthetic images. In this work, 50 image pairs were used randomly to measure the MS-SSIM score. In addition, PSNR denotes the ratio between the maximum intensity value to the present noise value. Greater value of PSNR indicates less amount of noise, which means the synthetic image has closer resemblance to the real image. We used 50 image pairs of real-to-projected images. We monitored the training process (i.e., training losses, FID score, and generated images) using Tensor Board to determine whether the StyleGAN2 was properly trained. The training took 64 h with two Tesla v100-sxm2-32 GB graphic processing units.

### Cancer detection with an anomaly detection method

**Figure 6:**
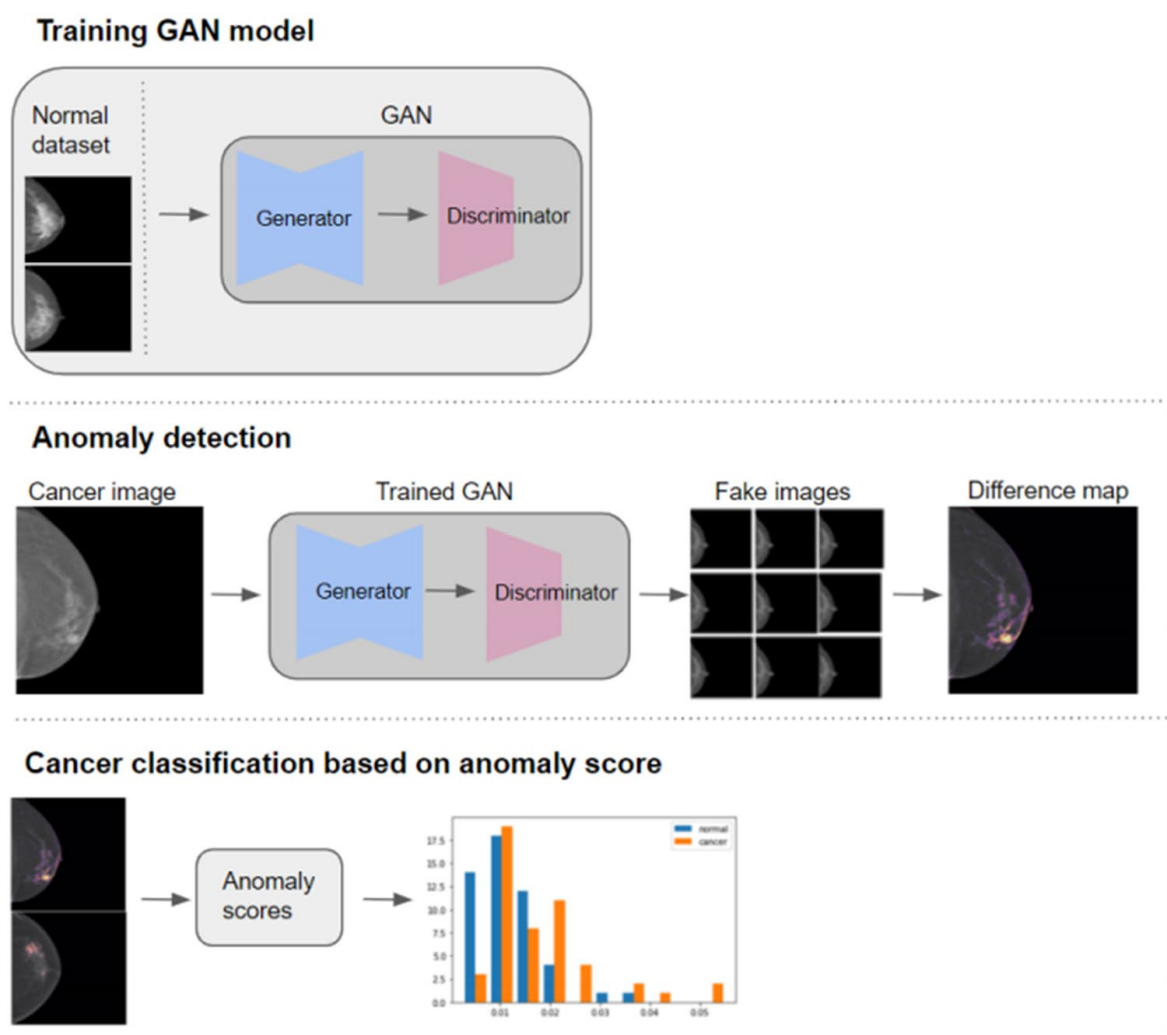
Workflow of classification for breast cancer using anomaly detection method.

Anomaly detection method aims to use a standard GAN, which was trained only on positive samples, to learn mapping from the latent space representation z to the realistic sample ${\hat{\text{x}}} = G(z)$, and use this learned representation to map unseen samples back to the latent space. Training GAN with normal samples alone makes the generator learn the manifold X of normal samples. Given that the generator learns how to generate normal samples, the difference between the input and the reconstructed image will highlight the anomalies. We yield normal synthetic images that were most similar to the test image with different seeds on the latent space of the StyleGAN2 by minimizing a perceptual loss. We tested 1, 9, and 16 seeds to find the optimal number of seeds to minimize the false-negative regions. For 9 and 16 seeds, one average image was obtained. Anomaly score was calculated by summing the difference maps between real and test images, which were then divided by the area of each breast. Finally, a threshold for the anomaly score that could classify normal and cancer images was determined by the threshold of the Youden J index. Figure 6 illustrates the overall workflow.

We evaluated the classification performance using AUC, accuracy, sensitivity, specificity, PPV, and NPV. The AUC was obtained to reflect the overall accuracy of the model. Equations (2)–(6) show the formulas of each metric used.

2 $$ {\text{Accuracy}} = \left( {TP + TN} \right)/\left( {TP + TN + FP + FN} \right) $$

3 $$ {\text{Sensitivity}} = TP/\left( {TP + FN} \right) $$

4 $$ {\text{Specificity}} = TN/\left( {TN + FP} \right) $$

5 $$ {\text{PPV}} = TP/\left( {TP + FP} \right) $$

6 $$ {\text{NPV}} = TN/\left( {TN + FN} \right) $$

## Data Availability

The datasets are not publicly available because of restrictions in the data-sharing agreements with the data sources. Ethics approval for the deidentified slides used in this study will be allowed upon request from the corresponding authors.
