# Publisher Correction: Unsupervised anomaly detection with generative adversarial networks in mammography

**DOI:** 10.1038/s41598-023-32395-w

**Published:** 2023-04-05

**Authors:** Seungju Park, Kyung Hwa Lee, Beomseok Ko, Namkug Kim

**Affiliations:** 1 Department of Biomedical Engineering, College of Health Sciences, Korea University, Seoul, Republic of Korea; 2 Department of Radiation Oncology, Korea University Guro Hospital, Korea University College of Medicine, Seoul, Republic of Korea; 3 Department of Breast Surgery, University of Ulsan College of Medicine and Asan Medical Center, 88 Olympic-ro 43-gil Songpa-gu, Seoul, 05505 Republic of Korea; 4 Department of Radiology, University of Ulsan College of Medicine and Asan Medical Center, Seoul, Republic of Korea; 5 Department of Convergence Medicine, Research Institute of Radiology and Institute of Biomedical Engineering, University of Ulsan College of Medicine and Asan Medical Center, 88 Olympic-ro 43-gil Songpa-gu, Seoul, 05505 Republic of Korea

Correction to: *Scientific Reports* https://doi.org/10.1038/s41598-023-29521-z, published online 20 February 2023

The original version of this Article contained an error in the Author Information section.

“These authors contributed equally: Seungju Park, Kyung Hwa Lee, Beomseok Ko and Namkug Kim.”

now reads:

“These authors contributed equally: Seungju Park and Kyung Hwa Lee.”

The original Article has been corrected.

